# Safety, pharmacokinetics and pharmacodynamics of ICP-332, an oral TYK2 inhibitor, in healthy participants: a randomized, double-blind, placebo-controlled phase I study

**DOI:** 10.3389/fphar.2026.1942461

**Published:** 2026-10-07

**Authors:** Hengli Zhao, Yilin Wei, Yu jiang, Kun He, Xiaoran Zhang, Rui Li, Qing Wen

**Affiliations:** Department of Clinical Research Center, Central Hospital Affiliated to Shandong First Medical University, Jinan, China

**Keywords:** C-reactive protein, food effect, ICP-332, pharmacodynamics, pharmacokinetics, TYK2 inhibitor

## Abstract

**Objective:**

ICP-332 is a novel, orally administered TYK2 inhibitor with high selectivity, intended for the treatment of immune inflammatory diseases such as atopic dermatitis, psoriatic arthritis, inflammatory bowel disease and lupus. This Phase I study assessed its safety, pharmacokinetics, pharmacodynamics, and food effect in Chinese healthy participants.

**Methods:**

This study was designed as a randomized, double-blind, placebo-controlled trial in healthy participants. It consisted of a single-ascending-dose (SAD) part with six cohorts (5, 15, 40, 80, 160, and 320 mg) and a multiple-ascending-dose (MAD) part with three cohorts (40, 80, and 160 mg administered once daily for 14 days). Food-effect evaluation was included at the 80 mg SAD level. In each cohort, eight participants were randomized (6:2) to receive either ICP-332 or placebo. Following oral administration, plasma samples were collected to determine ICP-332 concentrations, and safety tolerance was monitored and assessed throughout the study.

**Results:**

This study enrolled 72 healthy participants, with 48 and 24 completing the SAD and MAD phases, respectively. After oral administration, ICP-332 showed rapid absorption, with median time to peak concentration (*T*_max_) of 0.5–2.0 h and mean elimination half-life (*t*_*½*_) of 2.692–7.275 h. Dose-proportional exposure was confirmed in both the SAD (5–320 mg) and MAD (40–160 mg) phases; area under the concentration–time curve (*AUC*_*last*_) ranged from 146.3 to 11,667.5 h·ng/mL in the SAD phase and from 1386.0 to 6513.5 h·ng/mL at steady state in the MAD phase. ICP-332 also induced rapid and dose-dependent suppression of TYK2 activity, as evidenced by dose-related reductions in high-sensitivity C-reactive protein and a trend toward decreased interferon gamma-induced protein 10 levels *versus* placebo. In terms of safety, treatment-emergent adverse events were all mild to moderate in severity, with no participant withdrawals. TEAE incidence was comparable to placebo in the SAD phase and slightly higher in the MAD phase.

**Conclusion:**

ICP-332 exhibited favorable safety, pharmacokinetics and pharmacodynamics profiles across the 5–320 mg dose range, with rapid absorption, dose-proportional exposure, and mild, reversible adverse events. A high-fat meal did not significantly affect its pharmacokinetics, pharmacodynamics or safety. These results support continued clinical development of ICP-332 as a promising treatment for autoimmune diseases.

**Clinical Trial Registration:**

http://www.chinadrugtrials.org.cn/index.html CTR20211781.

## 1 Introduction

Autoimmune diseases are disorders characterized by immune responses directed against self-antigens, resulting in inflammation and damage to specific organs, tissues, and cells. These diseases are generally chronic, with a clinical course marked by recurrent exacerbations and the potential for significant disability or fatality (Miller, 2023). Currently, due to the lack of curative therapies, clinical management for autoimmune diseases primarily emphasizes symptomatic relief and inhibition of disease progression to minimize cumulative target organ injury (Koumpouras and Caricchio, 2026; Usmani et al., 2026). The therapeutic landscape for autoimmune diseases has advanced with the introduction of oral small-molecule targeted agents, particularly Janus kinase (JAK) inhibitors, that achieve selective immunosuppression and are deemed more appropriate for long-term administration (Jamilloux et al., 2019). Despite the success of several marketed agents in treating conditions, such as rheumatoid arthritis and inflammatory bowel disease, their clinical utility remains constrained by safety liabilities attributable to off-target effects. This gap in research underscores an urgent need to develop next-generation oral small-molecule therapeutics with superior selectivity and favorable safety windows.

The JAK Signal Transducer and Activator of Transcription (STAT) signaling pathway is a crucial immune-related signal transduction system that mediates cellular responses stimulated by various cytokines. JAK, a key component of this pathway, belongs to the family of intracellular nonreceptor tyrosine kinases. In mammals, the JAK family is comprised of four members: JAK1, JAK2, JAK3, and TYK2. Among them, TYK2 plays a significant role in the intracellular signaling of cytokines, such as type I interferons, IL-6, IL-10, IL-12, and IL-23 (Krueger et al., 2022). These signaling cascades contribute to the activation of Th17, Th1, B lymphocytes and myeloid cells, thereby modulating immune and inflammatory responses. Accordingly, dysregulation of these processes is implicated in the pathogenesis of various autoimmune and chronic inflammatory diseases (Yasuda et al., 2019; Bekić and Tomić, 2023).

The pan-JAK inhibitor was the first small-molecule JAK inhibitor to be successfully developed. Although it has demonstrated considerable therapeutic efficacy, it is associated with safety concerns, including the risk of reduced red blood cell and platelet production due to JAK2 inhibition (O'Sullivan et al., 2026; Zhu et al., 2026) and the potential for infections and carcinogenesis resulting from broad immunosuppression. In contrast, selective TYK2 inhibitors are expected to overcome these limitations by specifically targeting the type I interferon, IL-12, and IL-23 signaling pathways, thereby resulting in more precise therapeutic effects. Although no TYK2 inhibitor has received global approval for clinical use to date, several candidates—such as B-165 (Papp et al., 2018; Mease et al., 2022), P-067 (Forman et al., 2020; Page et al., 2020), and P-068 (Tehlirian et al., 2022; Morand et al., 2024)—have progressed to clinical trials and have shown promising efficacy and favorable tolerability in several autoimmune diseases.

ICP-332, a novel oral TYK2 inhibitor, with potent inhibitory activity and high selectivity, exhibits an approximately 400-fold selectivity for TYK2 over JAK2, which is expected to mitigate the risk of anemia associated with JAK2 inhibition. This molecule has also demonstrated some activity against JAK1. However, its selectivity for TYK2 over JAK1 was approximately 40-fold more, highlighting its potential as a highly efficient and selective TYK2 inhibitor (Xu et al., 2026). ICP-332 selectively blocks TYK2-mediated tyrosine kinase activity by binding to the TYK2 JH1 catalytic domain, thereby disrupting the downstream signaling of key inflammatory cytokines, including type I interferons, IL-12, and IL-23. This mechanism ultimately suppresses the aberrant immune and inflammatory responses underlying diseases, such as atopic dermatitis, psoriasis, systemic lupus erythematosus, and inflammatory bowel disease, for which ICP-332 is intended to be developed. In this regard, we conducted a phase I study to assess the safety, pharmacokinetics (PK), pharmacodynamics (PD) and food effect of ICP-332 in healthy individuals.

## 2 Methods

### 2.1 Study population

Key inclusion criteria were: healthy male or infertile female subjects aged 18–65 years; body mass index between 18 and 30 kg/m^2^; and absence of clinically significant abnormalities on physical examination, vital signs, 12-lead electrocardiography, laboratory tests, and medical history.

Key exclusion criteria were: positive serological results for hepatitis B virus, hepatitis C virus, human immunodeficiency virus, or syphilis; a history of tuberculosis or any active/latent infection; presence of genetic immunodeficiency in a first-degree relative; receipt of systemic anti-infective therapy within 14 days before enrollment; recent or planned use of potent cytochrome P450 3A4 inhibitors or inducers; significant gastrointestinal disorders that could interfere with drug pharmacokinetics; or any other clinically significant condition deemed by the investigator.

### 2.2 Study design and treatment

This phase I, randomized, double-blind, placebo-controlled, single- and multiple-ascending-dose study evaluated the PK, PD and food effect of ICP-332 in healthy Chinese participants. The protocol and informed consent forms were approved by the Independent Ethics Committee of Jinan Central Hospital (Jinan, China). All participants provided written informed consent prior to enrollment. The trial adhered to the Declaration of Helsinki and the Chinese National Medical Products Administration’s Good Clinical Practice (GCP, 2020) guidelines. Study details are registered with the China Drug Trials website (CTR20211781), and the design is illustrated in Figure 1.

**FIGURE 1:**
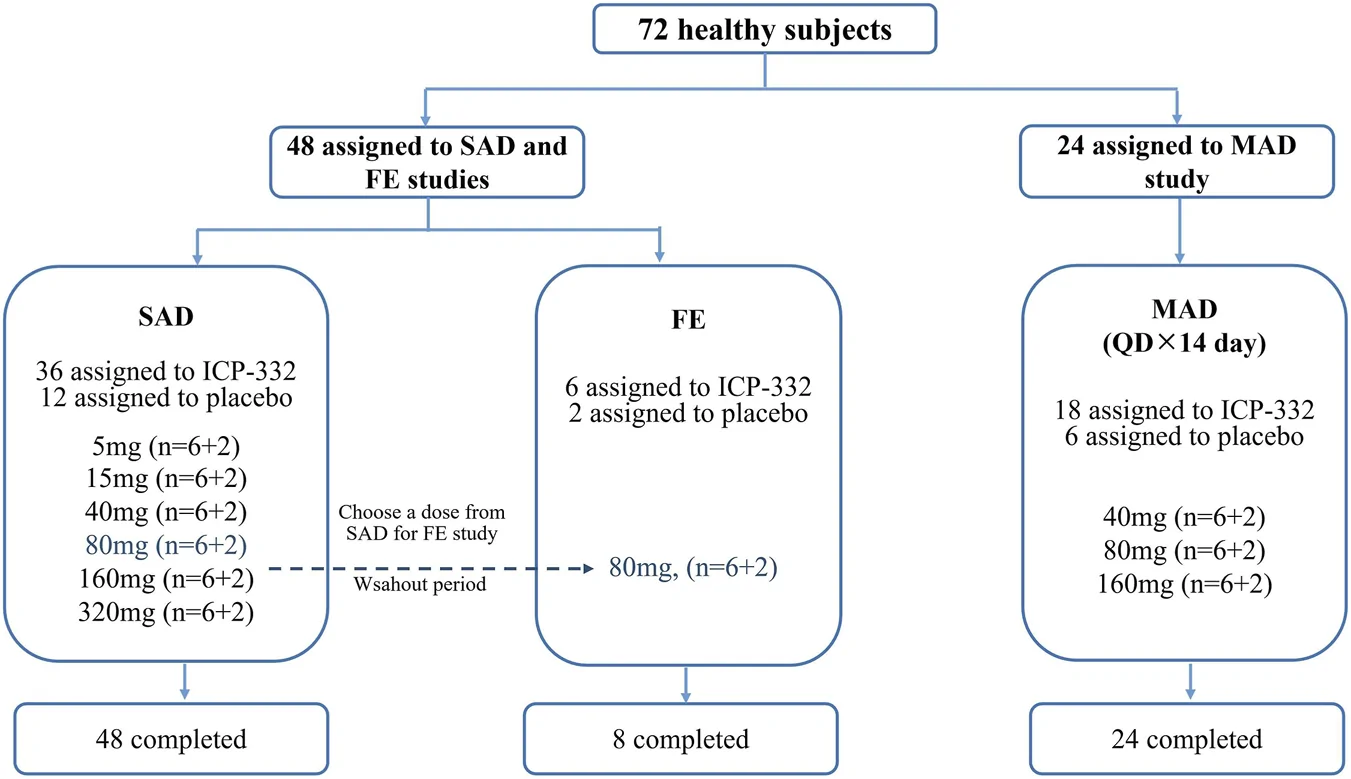
Schematic diagram of study design SAD, single ascending dose; MAD, multiple ascending dose; QD, Once a day.

#### 2.2.1 Single-ascending-dose and food effect study

The single-ascending-dose (SAD) part enrolled 48 healthy participants, who received a single oral dose of ICP-332 or matching placebo in a double-blind fashion. Each of the six dose cohorts comprised eight participants. A sentinel dosing strategy was implemented: the first two participants in each cohort were randomized 1:1 to receive either ICP-332 or placebo. Following a 48-h safety review, the remaining six participants were administered active drug or placebo at a 5:1 ratio. All doses were given under fasting conditions. Safety assessments continued through Day 4, with a follow-up visit on Day 8 and a final safety evaluation on Day 28. Dosing for the next cohort proceeded only after completion of at least the Day 8 follow-up visit, provided that the previous dose level was shown to be safely tolerated.

The food-effect (FE) cohort used the 80 mg dose, selected based on safety and PK data from the single-ascending-dose (SAD) part. Participants who had received the 80 mg dose in the SAD part underwent a 7-day washout, after which they were readmitted and given a single 80 mg dose of ICP-332 following a high-fat, high-calorie meal. The meal provided approximately 150 kcal from protein, 250 kcal from carbohydrates, and 500–600 kcal from fat, totaling 800–1000 kcal. Standardized meals were also provided at 4 h and 9–11 h post-dose on Day 1. Safety monitoring included intensive observation through Day 4, an end-of-study visit on Day 8, and a final safety follow-up on Day 28.

#### 2.1.2 Multiple-ascending-dose trials

Based on the SAD results, three dose levels (40, 80, and 160 mg) were selected for the multiple-ascending-dose (MAD) part. In each dose cohort, eight participants were randomized 3:1 to receive ICP-332 or placebo once daily under fasting conditions for 14 consecutive days. Safety assessments continued through Day 21, with an end-of-study visit on Day 28 and a final safety follow-up conducted at Day 28. Dosing for the next dose group was conducted after completion of the 14-day follow-up visit, provided that the previous dose group was safely tolerated.

### 2.3 Sample collection

In the SAD and FE parts, blood samples (3 mL each) for PK profiling were collected at pre-dose (0 h) and at 0.25, 0.5, 1, 2, 4, 6, 8, 12, 24, 36, 48, and 72 h post-dose. In the MAD part, PK samples (3 mL each) were collected on Days 1, 2, 6, 10, 14, 15, 16, and 17; on Days 1 and 14, the sampling times included pre-dose and at 0.25, 0.5, 1, 2, 4, 6, 8, and 12 h post-dose, while on the other days, trough samples were obtained within 30 min before dosing. All blood samples were drawn into EDTA tubes; plasma was separated by centrifugation and stored at −80 °C until analysis. Plasma concentrations of ICP-332 were determined using a validated high-performance liquid chromatography-tandem mass spectrometry (HPLC-MS/MS) method.

For pharmacodynamic assessments, additional blood samples were collected in the MAD part before the first dose and on Days 3, 7, 11, 15, 21, and 28 post-dose to evaluate JAK1 signaling activation. Serum was isolated for measuring interferon gamma-induced protein 10 (IP-10) and high-sensitivity C-reactive protein (hs-CRP), alongside routine hematological examinations.

### 2.4 Safety and tolerance evaluation

Safety assessments comprised laboratory tests, physical examinations, vital signs (pulse rate, blood pressure, body temperature, and respiratory rate), and 12-lead electrocardiography. All treatment-emergent adverse events (TEAEs) were recorded from the first dose through Day 28 after the last administration. Adverse events (AEs) were coded using the Medical Dictionary for Regulatory Activities (MedDRA) version 24.0 and graded per the Common Terminology Criteria for Adverse Events (CTCAE) version 5.0; their type, frequency, severity, and relationship to study treatment were documented.

### 2.5 Pharmacokinetic analysis

Plasma PK parameters of ICP-332 were determined using non-compartmental analysis with Phoenix WinNonlin (version 8.1; Pharsight Corporation, Mountain View, CA, USA). The following parameters were derived: area under the concentration-time curve from time zero to the last measurable concentration (*AUC*_*last*_), extrapolated AUC from time zero to infinity (*AUC*_*inf*_), apparent volume of distribution (*V*_*z*_ */F*), apparent clearance (*CL/F*), maximum plasma concentration (*C*_max_), and time to *C*_max_ (*T*_max_). Prior to statistical analysis, *AUC*_*last*_, *AUC*_*inf*_, *V*_*z*_ */F*, and *CL/F* were natural log-transformed. The accumulation index (R) for AUC over the dosing interval (*AUC*_*τ*_) and for *C*_max_ at steady state (ss) was calculated as follows: where *AUC*_*τ,ss*_ and *C*_max*,ss*_ are the values at steady state, and *AUC*_*τ,single*_ and *C*_max*, single*_ are the corresponding values after a single dose.

$$R_{AUC}=\frac{{AUC}_{\tau ,ss}}{{AUC}_{0-\tau ,single}}\text{ and }R_{C_{\max}}=\frac{C_{\max,ss}}{C_{\max,single}}$$

### 2.6 Statistical analysis

All analyses were conducted in the respective analysis populations. The safety set comprised all participants who received at least one dose of ICP-332 or placebo. The PK set included safety-set participants with at least one quantifiable plasma concentration of ICP-332, and the PD set included those with at least one valid PD measurement. Baseline characteristics, safety data, and PK/PD parameters were summarized using descriptive statistics. PK parameters were estimated *via* non-compartmental analysis. For the food-effect assessment, geometric means and 90% confidence intervals were calculated from log-transformed data. Statistical analyses were performed using SAS (version 9.4) and Phoenix WinNonlin.

## 3 Results

### 3.1 Participants

A total of 321 healthy participants were screened, of whom 72 eligible individuals were enrolled. Of these, 48 participants were assigned to SAD part (six dose cohorts; eight participants per cohort, randomized 6:2 to ICP-332 or placebo), and 24 were assigned to MAD part (three dose cohorts; eight participants per cohort, randomized 6:2 to ICP-332 or placebo). All enrolled participants completed the study as assigned. Demographic and baseline characteristics were generally balanced across dose groups and between active treatment and placebo, and are summarized in Table 1.

**TABLE 1:** Demographics of healthy participants.

Parameters (Unit)	SAD and FE							MAD			
	Placebo (N = 12)	5 mg (N = 6)	15 mg (N = 6)	40 mg (N = 6)	80 mg (N = 6)	160 mg (N = 6)	320 mg (N = 6)	Placebo (N = 6)	40 mg (N = 6)	80 mg (N = 6)	160 mg (N = 6)
Age, years	​	​	​	​	​	​	​	​	​	​	​
Mean (SD)	25.3 (7.2)	23.7 (4.6)	27.0 (7.0)	24.5 (4.1)	30.0 (3.8)	24.2 (4.5)	24.3 (4.1)	33.2 (5.5)	30.3 (8.3)	28.0 (5.0)	28.3 (5.0)
Range	18, 45	20, 31	19, 39	20, 30	24, 34	19, 30	18, 29	24, 39	20, 44	22, 34	23, 34
Sex, n (%)	​	​	​	​	​	​	​	​	​	​	​
Male	12 (100%)	6 (100%)	6 (100%)	6 (100%)	6 (100%)	6 (100%)	6 (100%)	6 (100%)	6 (100%)	6 (100%)	6 (100%)
Female	0	0	0	0	0	0	0	0	0	0	0
Height, cm	​	​	​	​	​	​	​	​	​	​	​
Mean (SD)	171.5 (5.5)	169.9 (8.2)	170.4 (8.8)	176.0 (7.4)	168.6 (5.3)	175.8 (4.6)	174.3 (3.1)	169.4 (4.8)	171.3 (3.8)	171.1 (6.8)	174.0 (7.0)
Range	162.5, 181.5	160.5, 180.5	158.5, 182.0	162.5, 184.0	163.0, 176.0	168.5, 181.0	170.5, 178.0	162.0, 175.5	165.5, 177.0	162.0, 178.5	168.0, 187.5
Weight, kg	​	​	​	​	​	​	​	​	​	​	​
Mean (SD)	65.4 (6.1)	64.6 (7.9)	64.2 (6.1)	68.4 (10.6)	61.5 (8.3)	71.4 (10.2)	66.0 (8.7)	64.5 (7.6)	68.3 (7.1)	67.0 (8.0)	72.8 (9.0)
Range	54.4, 79.7	57.8, 77.0	53.6, 71.2	55.4, 83.3	54.0, 76.4	56.5, 83.6	55.0, 76.7	53.8, 75.5	60.4, 79.6	53.4, 76.3	60.4, 87.4
BMI, kg/m^2^	​	​	​	​	​	​	​	​	​	​	​
Mean (SD)	22.2 (1.9)	22.4 (2.0)	22.2 (2.6)	22.0 (2.5)	21.6 (2.4)	23.1 (2.7)	21.7 (2.3)	22.4 (2.5)	23.2 (1.5)	22.9 (2.4)	24.0 (1.9)
Range	18.4, 25.7	19.8, 25.1	18.8, 25.7	18.1, 25.9	19.1, 24.7	18.9, 25.9	18.3, 24.2	19.5, 25.9	21.6, 25.4	18.6, 25.7	20.4, 25.7

Data are mean (standard deviation) and range (minimum, maximum) for all parameters; *N*, number of participants in full analysis set; *BMI*, body mass index.

### 3.2 Pharmacokinetics

#### 3.2.1 SAD

Thirty-six healthy participants received single oral doses of 5, 15, 40, 80, 160, or 320 mg of ICP-332 under fasting conditions. The mean plasma concentration-time profiles are presented in Figure 2, and the corresponding PK parameters, derived from ICP-332 plasma concentrations, are summarized in Table 2.

**FIGURE 2:**
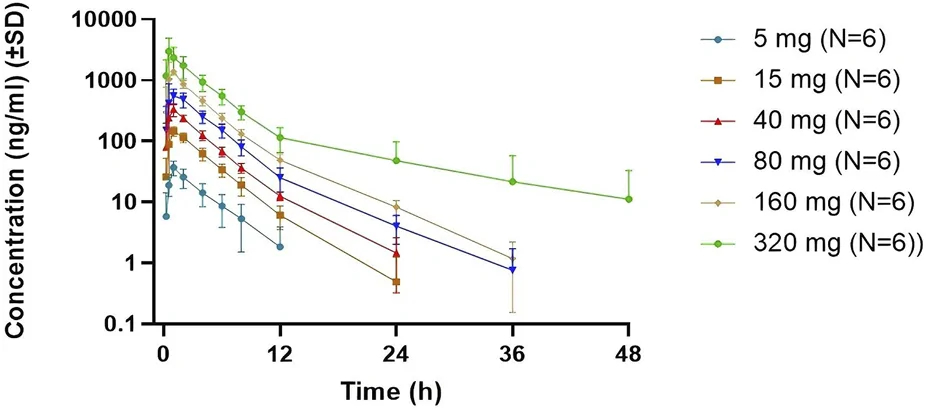
Mean concentration-time semilogarithmic curve for ICP-332 in plasma in SAD study.

**TABLE 2:** Pharmacokinetic parameters for ICP-332 in the SAD and FE study.

Parameters, unit	SAD						FE
	5 mg (N = 6)	15 mg (N = 6)	40 mg (N = 6)	80 mg (N = 6)	160 mg (N = 6)	320 mg (N = 6)	80 mg (N = 6)
*AUC*_*last*_, h·ng/mL	146.7 (71.6)	605.2 (120.9)	1297.7 (115.5)	2650.2 (579.0)	5083.7 (812.0)	11,667.5 (2021.4)	2493.5 (318.2)
*AUC*_*inf*_ *,* h·ng/mL	151.7 (72.3)	617.4 (118.3)	1317.5 (110.0)	2661.8 (578.1)	5104.8 (801.3)	11,757.2 (2074.4)	2509.2 (320.2)
*C*_max_, ng/mL	37.9 (8.6)	151.0 (23.6)	373.0 (80.0)	710.7 (267.5)	1596.7 (542.8)	3229.3 (1720.1)	707.5 (286.1)
*T*_max_, h	1.00 (0.50, 1.00)	1.00 (0.50, 1.00)	1.00 (0.50, 1.00)	1.00 (0.50, 2.00)	1.00 (0.50, 1.00)	1.00 (0.50, 2.00)	1.00 (1.00, 2.00)
*T*_*1/2*_, h	2.692 (0.903)	2.705 (0.796)	3.257 (0.818)	4.092 (1.151)	4.885 (1.106)	7.215 (4.302)	7.002 (5.565)
*CL/F*, L/h	37.2 (10.9)	25.0 (4.5)	30.5 (2.6)	31.3 (7.2)	32.0 (4.8)	28.0 (5.5)	32.3 (4.1)
*V*_*z*_ */F*, L	133.5 (22.3)	94.4 (18.2)	143.0 (36.7)	178.2 (34.4)	221.2 (36.6)	292.2 (196.3)	314.6 (218.3)

Data are median (range) for *T*_max_ and mean (standard deviation) for other parameters.

*AUC*_*last*_ area under the concentration–time curve from zero to the last measurable concentration, *AUC*_*inf*_, area under the concentration–time curve from zero extrapolated to infinity, *C*_max_ maximum serum concentration, *T*_*1/2*_ elimination half-life, *T*_max_ time to reach maximum plasma concentration, *CL/F* apparent clearance, *Vz/F* apparent volume of distribution.

Following single-dose administration, ICP-332 was rapidly absorbed, with median *T*_max_ values ranging from 0.5 to 2.0 h across all dose groups. Mean *C*_max_ and *AUC*_*last*_ increased in an approximately dose-proportional manner across the 5–320 mg range. Dose-normalized parameters were comparable among dose levels, with no significant differences observed, supporting linear PK over the tested dose range (Supplementary Figure S1).

#### 3.2.2 FE

Participants from the SAD 80 mg cohort returned after a 7-day washout for the food-effect assessment. The mean plasma concentration-time profiles of ICP-332 under fasting and fed conditions are presented in Figure 3, with corresponding PK parameters summarized in Table 2.

**FIGURE 3:**
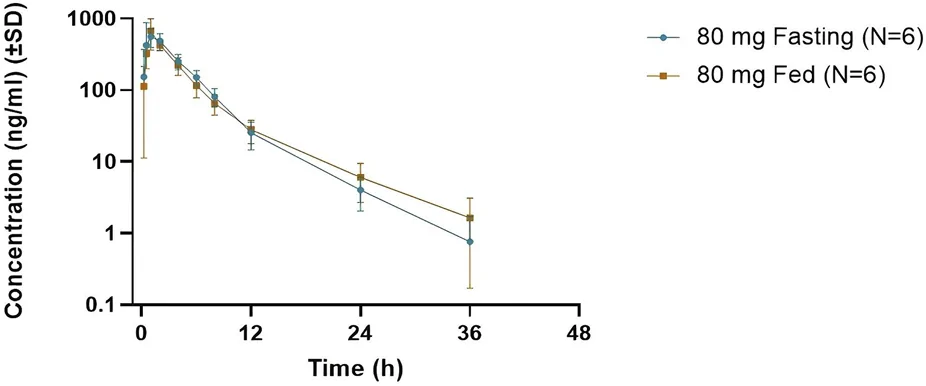
Mean concentration-time semilogarithmic curve for ICP-332 in plasma in FE study.

The geometric mean ratios (GMRs, fed/fasted) for *C*_max_ and *AUC*_*last*_ were 0.99 and 0.95, respectively. The 90% confidence intervals for the GMRs of *AUC*_*last*_ and *AUC*_*inf*_ fell entirely within the 80.00%–125.00% bioequivalence bounds. These findings indicate that administration of a single 80 mg oral dose of ICP-332 with a high-fat, high-calorie meal did not significantly alter its plasma exposure, suggesting that food has no clinically relevant effect on the PK profile of ICP-332.

#### 3.2.3 MAD

Eighteen healthy participants received 14 consecutive daily doses of 40, 80, or 160 mg ICP-332. The mean plasma concentration-time profiles on Day 1 and Day 14 are shown in Figure 4, with corresponding PK parameters summarized in Table 3.

**FIGURE 4:**
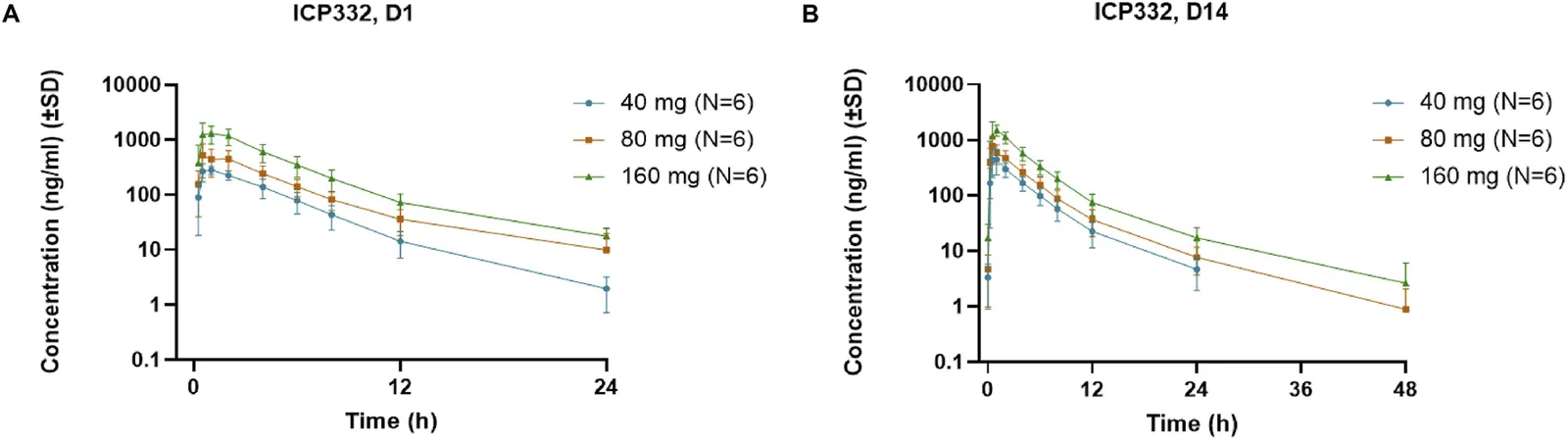
Mean concentration-time semilogarithmic curve for ICP-332 in plasma in MAD study. **(A)** Curve for the first dose of ICP-332; **(B)** Curve for the last dose of ICP-332.

**TABLE 3:** Pharmacokinetic parameters for ICP-332 in the MAD study.

Parameters, unit	D1			D14		
	40 mg (N = 6)	80 mg (N = 6)	160 mg (N = 6)	40 mg (N = 6)	80 mg (N = 6)	160 mg (N = 6)
*AUC*_*last*_, h*ng/mL	1359.6 (371.5)	2600.0 (777.1)	6503.0 (1559.7)	1918.2 (551.5)	3034.0 (828.8)	6705.7 (1752.1)
*AUC*_*inf*_, h*ng/mL	1370.6 (374.6)	2708.2 (791.5)	6618.8 (1564.4)	1942.8 (558.7)	3060.2 (830.0)	6745.5 (1742.3)
*C*_max_, ng/mL	318.2 (47.3)	659.3 (210.2)	1781.7 (252.7)	529.8 (190.0)	898.5 (212.5)	1715.0 (569.8)
*C*_*ss_av*_, ng/mL	—	—	—	79.2 (23.2)	123.6 (32.3)	270.2 (66.6)
*T*_max_, h	0.75 (0.50, 2.00)	0.75 (0.50, 2.00)	0.75 (0.50, 2.00)	0.75 (0.50, 2.00)	0.50 (0.50, 2.00)	1.00 (0.50, 2.00)
*T*_*1/2*_, h	3.105 (0.625)	5.477 (2.683)	4.470 (1.114)	4.787 (2.085)	5.373 (1.814)	7.275 (3.822)
*CL/F*, L/h	31.3 (9.3)	32.8 (14.2)	25.4 (6.2)	22.4 (5.8)	29.5 (11.6)	25.9 (6.3)
*V*_*z*_ */F*, L	134.5 (27.5)	262.3 (164.5)	169.7 (83.3)	156.2 (82.1)	209.6 (38.7)	263.7 (134.7)

Data are median (range) for *T*_max_ and mean (standard deviation) for other parameters. *AUC*_*last*_ area under the concentration–time curve from zero to the last measurable concentration, *AUC*_*inf*_, area under the concentration–time curve from zero extrapolated to infinity, *C*_max_ maximum serum concentration, *T*_*1/2*_ elimination half-life, *T*_max_ time to reach maximum plasma concentration, *CL/F* apparent clearance, *Vz/F* apparent volume of distribution, *C*_*ss_av*_ average steady-state plasma concentration.

Following multiple dosing, ICP-332 was rapidly absorbed, with median *T*_max_ on Day 14 ranging from 0.5 to 2.0 h post-dose. Elimination occurred at a comparable rate across dose groups, with a mean terminal half-life (*t*_*1/2*_) of 4.8–7.3 h. Mean *C*_max_ and *AUC*_*last*_ increased in a dose-proportional manner over the 40–160 mg range, as supported by the lack of significant differences in dose-normalized parameters among the three dose groups (Supplementary Figure S2). No clinically meaningful accumulation was observed after 14 days of once-daily dosing; the accumulation ratios (*R*_*AUC*_ and *R*_*Cmax*_) across the 40–160 mg dose range ranged from 1.022 to 1.467 and 0.992 to 1.668, respectively.

### 3.3 Pharmacodynamics

In the MAD part, ICP-332 was administered once daily for 14 consecutive days. Pharmacodynamic assessments, including hs-CRP and routine hematological parameters, were monitored through Day 28.

#### 3.3.1 hs-CRP

Multiple-dose administration of ICP-332 produced a dose-dependent suppression of serum hs-CRP levels (Table 4). Significant reductions from baseline were observed following treatment, with maximal suppression occurring between Day 11 and Day 15 in the 40, 80, and 160 mg dose groups. However, the 40 mg group exhibited an atypical pattern during this period. After Day 15, hs-CRP levels gradually rebounded toward baseline by Day 21 and approached baseline levels by Day 28. Of note, one subject in the 40 mg group experienced an upper respiratory tract infection on Day D12, which likely contributed to the markedly elevated standard deviation in hs-CRP levels observed on Day 15 in this dose group.

**TABLE 4:** Percentage changes of hs-CRP and IP-10 from baseline in the MAD study.

Visit	hs-CRP (%)				IP-10 (%)			
	Placebo (N = 6)	40 mg (N = 6)	80 mg (N = 6)	160 mg (N = 6)	Placebo (N = 6)	40 mg (N = 6)	80 mg (N = 6)	160 mg (N = 6)
Day 3	​	​	​	​	​	​	​	​
Mean (SD)	−11.4 (42.7)	−47.5 (26.0)	−44.6 (25.5)	−13.1 (52.1)	24.0 (38.8)	9.5 (11.3)	−4.7 (29.2)	955.4 (1585.4)
Range	−62.5, 40.0	−75.0, 0	−75.0, 0	−62.5, 75.0	−28.6, 62.8	−2.8, 27.9	−47.2, 36.9	−32.3, 3658.9
Day 7	​	​	​	​	​	​	​	​
Mean (SD)	37.8 (75.4)	−62.6 (20.1)	−65.0 (32.1)	−12.7 (83.4)	68.9 (70.9)	−1.3 (12.8)	−4.4 (37.5)	160.8 (334.6)
Range	−40.0, 150.0	−85.0, −33.3	−81.3, 0	−81.3, 125.0	−55.5, 133.8	−13.5, 18.1	−67.4, 39.0	−28.9, 819.6
Day 11	​	​	​	​	​	​	​	​
Mean (SD)	11.9 (35.4)	−22.8 (59.1)	−49.4 (26.8)*	−65.2 (32.2)*	33.5 (58.0)	3.3 (12.3)	6.9 (37.3)	38.5 (82.6)
Range	−33.3, 50.0	−85.0, 50.0	−75.0, 0	−81.3, 0	−59.9, 109.3	−7.2, 24.1	−52.3, 52.5	−30.0, 183.9
Day 15	​	​	​	​	​	​	​	​
Mean (SD)	31.5 (71.6)	272.8 (532.0)	−52.2 (28.7)*	−61.9 (39.4)*	42.6 (71.7)	55.8 (127.4)	−9.7 (43.7)	−3.9 (31.6)
Range	−40.0, 150.0	−85.0, 1240.0^**^	−81.3, 0	−93.2, 0	−64.6, 142.9	−13.7, 313.2	−79.6, 53.7	−38.2, 53.6
Day 21	​	​	​	​	​	​	​	​
Mean (SD)	90.6 (114.8)	633.1 (1152.0)	190.9 (251.4)	138.4 (121.0)	9.1 (42.1)	63.9 (20.3)	26.4 (32.3)	30.2 (36.2)
Range	−16.7, 300.0	−40.0, 2960.0^**^	50.0, 700.0	−50.0, 275.0	−62.7, 61.5	37.0, 81.9	−32.6, 54.2	−15.6, 89.0
Day 28	​	​	​	​	​	​	​	​
Mean (SD)	72.9 (129.5)	157.5 (192.9)	215.9 (153.6)	79.9 (131.5)	7.8 (34.6)	63.6(81.3)	56.1 (42.7)	36.5 (42.8)
Range	−87.5, 300.0	0, 540.0	25.0, 433.3	−25.0, 325.0	−40.6, 58.9	5.6, 221.5	21.2, 114.6	−12.2, 97.1

Data are mean (standard deviation) and range (minimum, maximum) for all parameters.

* *p*-value <0.05, compared with Placebo group.

** hs-CRP, level for one participant in 40 mg group showed increased on D15, which might be related to the occurrence of upper respiratory tract infection on D12.

*hs-CRP:* high-sensitivity C-reactive protein; *IP-10*: Interferon-induced protein 10.

#### 3.3.2 IP-10

Compared with placebo, ICP-332 administration was associated with a tendency toward decreased serum IP-10 levels in the 40 and 80 mg dose groups (Table 4). In the 80 mg group, this reduction was observed as early as Day 3 and persisted through Day 15, whereas in the 40 mg group, it was only noted at Day 7. No consistent pattern was discernible in the 160 mg group, largely due to substantial inter-individual variability: two participants with intercurrent upper respiratory tract infections exhibited markedly elevated IP-10 levels, which likely masked the dose-dependent effect. Following treatment cessation, IP-10 levels returned to baseline across all dose groups.

#### 3.3.3 Blood cells

After 14 days of once-daily ICP-332 administration, no significant changes from baseline were observed in erythrocyte, platelet, or hemoglobin levels across any dose group (Figure 5). In 160 mg group, neutrophil counts demonstrated a declining tendency during the continuous dosing period and gradually returned to baseline levels between Days 21 and 28. Reticulocyte counts showed no significant changes in the 40 mg and 80 mg dose groups, but decreased in the 160 mg dose group. Nevertheless, their absolute values remained within the normal range and gradually recovered to baseline after treatment cessation. Lymphocyte counts did not show notable changes across any of the dose groups. This finding is consistent with JAK1 inhibition and serves as both a safety biomarker and an indicator of target engagement.

**FIGURE 5:**
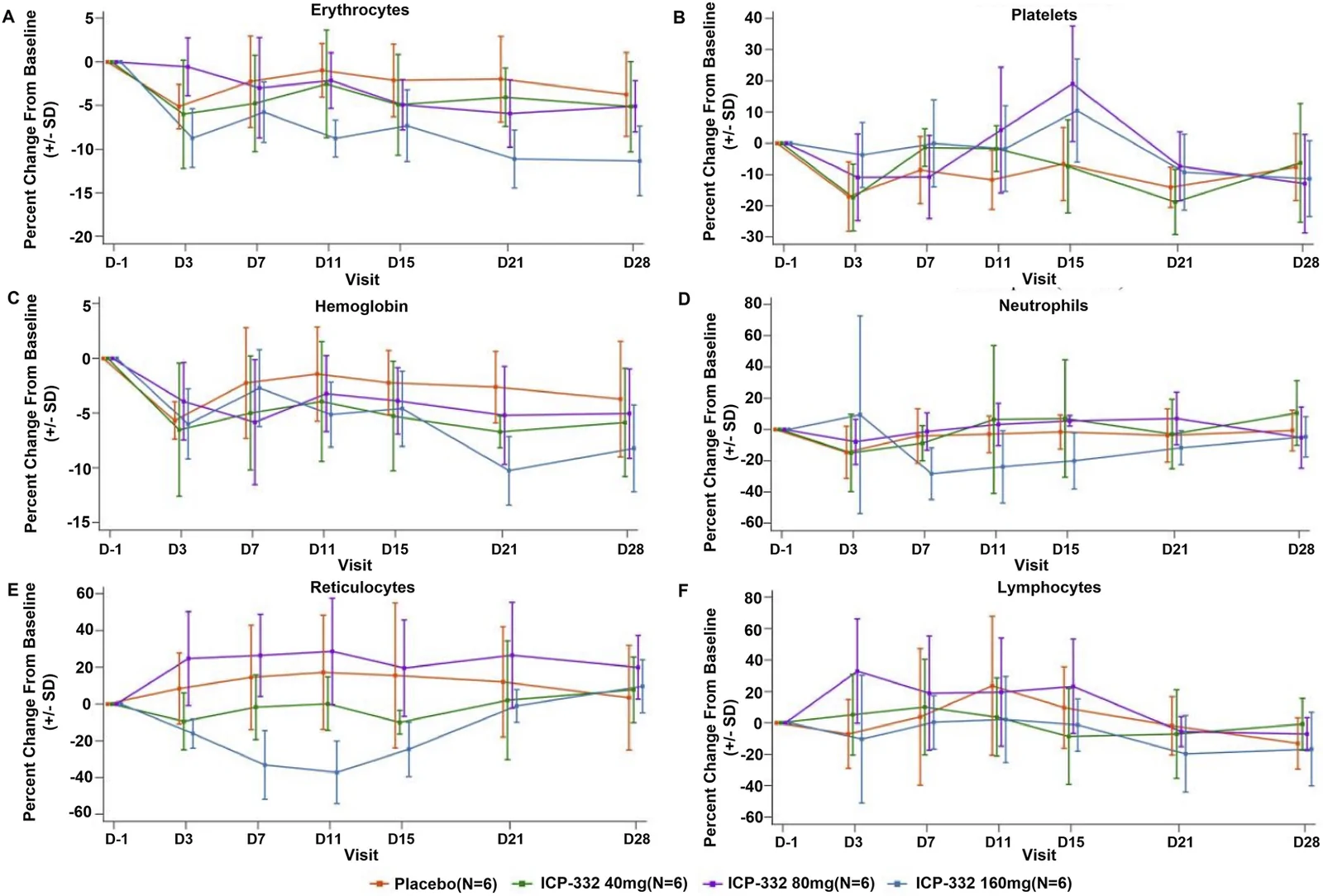
Mean percent change from baseline *versus* time for blood cell count in MAD study. Curves for pharmacodynamic index of Erythrocytes **(A)**, Platelets **(B)**, Hemoglobin **(C)**, Neutrophils **(D)**, Reticulocytes **(E)** and Lymphocytes **(F)**.

The observed reductions in hs-CRP and IP-10 establish these two cytokines as key pharmacodynamic biomarkers for ICP-332. Taken together, these findings support the high selectivity of ICP-332 for JAK1/TYK2 over JAK2, suggesting a potentially improved safety profile relative to less selective pan-JAK inhibitors.

### 3.4 Safety and tolerance

All 72 treated participants were included in the safety analysis, with outcomes summarized in Table 5 (SAD and FE) and Table 6 (MAD).

**TABLE 5:** Adverse events reported in the SAD and FE study.

AE type	SAD							FE		​
	placebo (N = 12)	5 mg (N = 6)	15 mg (N = 6)	40 mg (N = 6)	80 mg (N = 6)	160 mg (N = 6)	320 mg (N = 6)	placebo (N = 2)	ICP-332 (N = 6)	Total (N = 48)
All TEAEs	7 (58.3)	5 (83.3)	4 (66.7)	5 (83.3)	4 (66.7)	1 (16.7)	5 (83.3)	1 (50.0)	3 (50.0)	31 (64.6)
Mild	7 (58.3)	5 (83.3)	4 (66.7)	5 (83.3)	4 (66.7)	1 (16.7)	5 (83.3)	1 (50.0)	3 (50.0)	31 (64.6)
Moderate	0	0	0	0	0	0	0	0	0	0
Severe	0	0	0	0	0	0	0	0	0	0
All TRAEs	3 (25.0)	4 (66.7)	2 (33.3)	2 (33.3)	4 (66.7)	1 (16.7)	5 (83.3)	1 (50.0)	3 (50.0)	22 (45.8)
Mild	3 (25.0)	4 (66.7)	2 (33.3)	2 (33.3)	4 (66.7)	1 (16.7)	5 (83.3)	1 (50.0)	3 (50.0)	22 (45.8)
Moderate	0	0	0	0	0	0	0	0	0	0
Severe	0	0	0	0	0	0	0	0	0	0
Common TEAEs	​	​	​	​	​	​	​	​	​	​
Decreased plasma fibrinogen	3 (25.0)	4 (66.7)	0	2 (33.3)	4 (66.7)	0	3 (50.0)	1 (50.0)	3 (50.0)	17 (35.4)
Increased plasma triglycerides	1 (8.3)	2 (33.3)	1 (16.7)	0	0	1 (16.7)	2 (33.3)	0	0	7 (14.6)
Increased blood uric acid	3 (25.0)	0	0	1 (16.7)	1 (16.7)	1 (16.7)	0	0	0	6 (12.5)
Positive white blood cells in urine	0	0	1 (16.7)	2 (33.3)	0	0	1 (16.7)	0	0	4 (8.3)
Positive fungal test	1 (8.3)	0	2 (33.3)	0	0	0	0	0	0	3 (6.3)
Upper respiratory infection	0	1 (16.7)	0	0	0	0	0	0	1 (16.7)	2 (4.2)

Data are n (%), n, number of participants of each factor under each category. TEAE, treatment-emergent adverse event; TRAE, treatment-related adverse event; Common TEAEs, are TEAEs, reported by ≥ 2 participants in the ICP-332, or placebo group.

**TABLE 6:** Adverse events reported in the MAD study.

AE type	Placebo (N = 6)	40 mg (N = 6)	80 mg (N = 6)	160 mg (N = 6)	Total (N = 24)
All TEAEs	4 (66.7)	5 (83.3)	6 (100)	6 (100)	21 (87.5)
Mild	4 (66.7)	5 (83.3)	6 (100)	6 (100)	21 (87.5)
Moderate	0	1 (16.7)	0	0	1 (4.2)
Severe	0	0	0	0	0
All TRAEs	3 (50.0)	4 (66.7)	6 (100)	6 (100)	19 (79.2)
Mild	3 (50.0)	4 (66.7)	6 (100)	6 (100)	19 (79.2)
Moderate	0	1 (16.7)	0	0	1 (4.2)
Severe	0	0	0	0	0
Common TEAEs	​	​	​	​	​
Decreased plasma fibrinogen	1 (16.7)	4 (66.7)	6 (100)	6 (100)	17 (70.8)
Upper respiratory infection	0	1 (16.7)	1 (16.7)	2 (33.3)	4 (16.7)
Increased plasma triglycerides	1 (16.7)	0	2 (33.3)	0	3 (12.5)
Nausea	0	0	1 (16.7)	2 (33.3)	3 (12.5)
Positive white blood cells in urine	1 (16.7)	1 (16.7)	0	0	2 (8.3)
Positive red blood cells in urine	0	0	1 (16.7)	1 (16.7)	2 (8.3)
Positive urine occult blood	0	0	0	2 (33.3)	2 (8.3)

Data are n (%), n, number of participants of each factor under each category.

In the SAD and FE trials, TEAEs were reported in 24/36 (66.7%) participants in the ICP-332 group and 7/12 (58.3%) in the placebo group, and all were mild in severity (Table 5). The most common TEAEs (incidence >10%) were decreased fibrinogen (17/36, 35.4%), increased triglycerides (7/36, 14.6%), and increased uric acid (6/36, 12.5%). Incidences of decreased fibrinogen (36.1% vs. 25.0%) and increased triglycerides (16.7% vs. 8.3%) were higher in the ICP-332 group than in placebo, though no dose-dependent trend was observed. The high-fat meal did not appreciably affect the TEAE profile of ICP-332.

In the MAD part, TEAEs were reported in 17/18 (94.4%) participants in the ICP-332 group and 4/6 (66.7%) in the placebo group, with most being mild (Table 6). Common TEAEs included decreased fibrinogen (17/18, 94.4%), increased triglycerides (3/18, 12.5%), upper respiratory tract infection (URTI; 4/18, 16.7%), and nausea (3/18, 12.5%). Compared with placebo, the ICP-332 group had higher incidences of increased triglycerides (88.9% vs. 16.7%) and URTI (22.2% vs. 0%).

All TEAEs were mild in severity, except for one moderate URTI in the MAD 40 mg group. All affected participants were followed up and confirmed to have fully recovered, with no withdrawals due to AEs. Overall, ICP-332 was well tolerated in this study.

## 4 Discussion

TYK2 mediates downstream signaling of pro-inflammatory cytokines including IL-6, IL-12, and IL-23, making it an attractive therapeutic target for autoimmune diseases (Forman et al., 2020). Although TYK2 inhibitors have shown promising efficacy in preclinical models (Zhang et al., 2021; Mease et al., 2022), the development of highly selective inhibitors targeting the catalytically active JH1 domain has remained challenging (Chang, Xu et al., 2019). ICP-332 is a potent TYK2 inhibitor with approximately 400-fold selectivity over JAK2, a feature expected to minimize the risk of JAK2-mediated hematological toxicity (e.g., anemia). Our research results also indicated stable levels of erythrocytes, platelets, and hemoglobin in the participants following 14 consecutive administration of ICP-332 within the range of 40–160 mg, further confirming the high selectivity of ICP-332 for JAK2.

Following single or multiple oral doses, ICP-332 was rapidly absorbed and eliminated in healthy participants. *C*_max_ and *AUC*_*last*_ demonstrated dose-proportional pharmacokinetics over the 5–320 mg range. After 14 days of once-daily dosing (40–160 mg), no significant drug accumulation was observed, and a high-fat meal did not appreciably alter the PK profile of ICP-332. Collectively, these favorable PK properties—linear exposure, minimal accumulation, and food-independent absorption—support convenient once-daily oral dosing without meal restrictions in future clinical studies. A notable pharmacokinetic limitation is the compound’s rapid metabolic clearance. However, rapid clearance does not undermine efficacy, as TYK2 inhibitor activity is PD-driven rather than plasma-concentration-driven. PD biomarkers (IP-10, hsCRP, TARC) from the phase 2 trial confirm sustained target inhibition beyond drug circulation, consistent with slow off-rate kinetics (Liu et al., 2024; Xu et al., 2026). Thus, rapid clearance minimizes accumulation and off-target risks without compromising efficacy—supporting a favorable benefit-risk profile.

In this study, parameter *t*_*1/2*_ of ICP-332 was found to be significantly prolonged after the SAD group dosage was increased to 320 mg, and the elimination phase of the pharmacokinetic curve exhibited marked changes compared with that in the low-dose group. According to the literature, multiple cytochrome P450 enzymes are involved in the metabolism of compounds similar to ICP-332 (Dowty et al., 2024; Greenzaid and Feldman, 2024), leading us to hypothesize that ICP-332 may share a comparable metabolic pathway. Furthermore, at higher doses, the metabolic capacity of hepatic enzymes might approach saturation, resulting in downregulated overall metabolism and consequently an extended drug half-life. Furthermore, a drug-drug interactions clinical trial is currently conducted in our research group, and the forthcoming findings are expected to provide a more comprehensive insight into this phenomenon.

A dose-dependent pharmacodynamic response was observed in the MAD part. After 14 days of once-daily dosing, ICP-332 induced a dose-dependent reduction in serum hs-CRP relative to placebo, as well as a decreasing trend in IP-10 at the 40 and 80 mg doses. Both biomarkers returned toward baseline following treatment cessation. Given that hs-CRP and IP-10 expression is regulated by JAK1/STAT1 pathway signaling (Febvre-James et al., 2020; Wang et al., 2023), these findings support their utility as pharmacodynamic biomarkers of ICP-332-mediated JAK1 inhibition.

In the 160 mg group, a transient decrease in neutrophil counts was observed during treatment. This finding is consistent with JAK1/TYK2 pathway inhibition, as cytokines involved in neutrophil regulation—including IL-6, IL-12, and IL-10—signal through JAK1 and TYK2 to activate STAT pathways (Milara et al., 2022; Kuley et al., 2025). In contrast, erythrocyte, platelet, and hemoglobin levels remained stable across all dose groups. Reticulocyte counts showed a mild, transient decrease only in the 160 mg group, with values remaining within the normal range and recovering after treatment cessation. Given that JAK2 mediates erythropoiesis and thrombopoiesis *via* erythropoietin and thrombopoietin receptors (Nogueira et al., 2020; Abraham et al., 2024), the minimal impact on these hematological parameters supports the high selectivity of ICP-332 for JAK1/TYK2 over JAK2. Collectively, these data confirm targeted JAK1/TYK2 inhibition and suggest a potentially improved safety profile relative to pan-JAK inhibitors.

Overall, ICP-332 was safe and well-tolerated in healthy participants at single doses up to 320 mg and multiple doses up to 160 mg once daily for 14 days. Most AEs were mild in severity, with no serious AEs or treatment-related discontinuations. In both the SAD and MAD parts, the most frequently reported treatment-related adverse event (TRAE) by preferred term was decreased serum fibrinogen. These changes were transient and resolved spontaneously without intervention by the end of the study.

Notably, this finding has not been commonly reported in clinical studies of other TYK2 inhibitors, and its underlying mechanism remains unclear. Fibrinogen deposition is a recognized hallmark of tissue injury and inflammation—including immune-mediated processes—and plays a central role in autoimmune disease pathogenesis (Luyendyk et al., 2019). ICP-332 inhibits TYK2 activity while also exerting moderate suppression on the broader JAK pathway. Given that JAK inhibitors are known to downregulate inflammatory signaling cascades and consequently reduce fibrinogen levels (Hansildaar et al., 2024), we examined this parameter in our study. Indeed, following multiple doses of ICP-332, the incidence of decreased fibrinogen levels was notably higher in the treatment group than in the placebo group. Importantly, all such events were mild in severity, which maybe attributed to the ICP-332 modulatory effect on JAK signaling.

However, given the small sample size per cohort in this early-phase trial, larger and longer-term studies are warranted to determine whether decreased fibrinogen represents a class effect of TYK2 inhibitors. In particular, investigations in patient populations with autoimmune diseases will be valuable to clarify the clinical relevance of this observation.

No hepatorenal toxicity was observed with ICP-332 in this study. Renal dysfunction, a known adverse effect of several TYK2 inhibitors under clinical development that typically presents as elevated serum creatinine (Gooderham et al., 2022; Martin, 2023), was not detected in any dose cohort. Comprehensive liver function monitoring—including alanine aminotransferase (ALT) and aspartate aminotransferase (AST)—revealed no drug-related abnormalities throughout the study period. The absence of significant hepatic or renal injury suggests a potentially favorable safety profile for ICP-332 relative to other TYK2 inhibitors, possibly attributable to its selective targeting. These findings are preliminary given the small sample size and require confirmation in larger studies; nonetheless, they support the favorable safety and tolerability profile of ICP-332 observed in this phase I trial.

## 5 Conclusion

ICP-332 was rapidly absorbed after oral administration and displayed dose-proportional pharmacokinetics over the 5–320 mg range. Minimal accumulation was observed after 14 days of once-daily dosing (40–160 mg), and food intake did not appreciably alter its PK profile. The dose-dependent suppression of hs-CRP and the decreasing trend in IP-10 are consistent with JAK1/TYK2 pathway inhibition, supporting their use as pharmacodynamic biomarkers. The favorable safety profile—characterized by mild, reversible AEs, no hepatorenal toxicity, and minimal impact on JAK2-mediated hematological parameters—suggests a potential advantage over non-selective pan-JAK inhibitors. Collectively, these findings support the continued clinical development of ICP-332 as a selective TYK2 inhibitor for autoimmune diseases.

## Data Availability

The original contributions presented in the study are included in the article/Supplementary Material, further inquiries can be directed to the corresponding authors.
