# Supplementary material for: Safety, pharmacokinetics and pharmacodynamics of ICP-332, an oral TYK2 inhibitor, in healthy participants: a randomized, double-blind, placebo-controlled phase I study
Source: Front Pharmacol. 2026 Oct 7;17:1942461. doi: 10.3389/fphar.2026.1942461 (PMC13711260; doi:10.3389/fphar.2026.1942461)
Supplement: Supplementary file 1 [file Supplementaryfile1.docx]

**Supplementary materials**

**Safety, pharmacokinetics and pharmacodynamics of ICP-332,
 an oral TYK2 Inhibitor, in healthy participants: a randomized, double-blind, placebo-controlled phase I study**

Hengli Zhao^1^, Yilin Wei^1^, Yu jiang^1^, Kun He^1^, Xiaoran Zhang^1^, Rui Li^1^, Qing Wen^1^*

1. Department of Clinical Research Center, Central Hospital Affiliated to Shandong First Medical University, Jinan 250013, China

***Corresponding author:** Prof. Qing Wen

Department of Clinical Research Center, Central Hospital Affiliated to Shandong First Medical University, Jinan 250013, China

E-mail address:  [qwen@email.sdfmu.edu.cn](mailto:hlzhao@email.sdfmu.edu.cn;)

ORCID: 0000-0002-6408-2370

**Figure S1. Dose-normalized PK parameters for ICP-332 in different SAD dose cohorts.** (A) Dose-normalized *C_max_* of ICP-332; (B) Dose-normalized *AUC_last_* of ICP-332.

**Figure S2. Dose-normalized PK parameters for ICP-332 in different MAD dose cohorts.** (A) Dose-normalized *C_max_* of ICP-332; (B) Dose-normalized *AUC_last_* of ICP-332.
